# Extraskeletal Myxoid Chondrosarcoma With Rhabdoid Features in a Pediatric Patient

**DOI:** 10.7759/cureus.86605

**Published:** 2025-06-23

**Authors:** Juan J Andrade Rojas, Maria J Lizardo, Alma D Hernández Pérez, G Gomez Garza, Celso T Corcuera Delgado

**Affiliations:** 1 Faculty of Medical Sciences, Universidad de Cuenca, Cuenca, ECU; 2 Pathology, Instituto Nacional de Pediatría, Mexico City, MEX; 3 Electronic Microscopy Laboratory, Instituto Nacional de Rehabilitación-Luis Guillermo Ibarra Ibarra, Mexico City, MEX; 4 Radiology, Instituto Nacional de Pediatría, Mexico City, MEX

**Keywords:** child, electronic microscopy, embryonal chondrosarcoma, extraskeletal myxoid chondrosarcoma, immunohistochemistry, rhabdoid

## Abstract

Extraskeletal myxoid chondrosarcoma is an aggressive tumor in children. Due to its low incidence and nonspecific clinical presentation, as well as its radiological and histopathological characteristics, it is considered a diagnostic challenge. Furthermore, despite the NR4A3 rearrangement being specific to this neoplasm, its evaluation is not routinely performed, as few places have the technology to characterize it. We present the case of a 12-year-old girl with extraskeletal myxoid chondrosarcoma in the right thigh associated with lung metastasis. The diagnosis was ultimately made by integrating the clinical, radiological, histopathological, and ultrastructural features of the chondroblastic differentiation.

## Introduction

Extraskeletal myxoid chondrosarcoma (EMC) is a soft tissue tumor composed of primitive cells with chondroid features embedded in a myxoid matrix. It most frequently affects the thighs and has a high rate of local relapse and metastasis [1,2]. It is the least frequent tumor among the Ewing sarcoma family and is characterized by rearrangement of the NR4A3 gene, which fuses with EWSR1 in 62 to 74% of cases, with a minority fusing with TAF15, TCF12, TFG, FUS, or HSPA8 [3].

Macroscopically, it is an encapsulated nodular tumor, usually measuring less than 10 cm, with a white gelatinous cut surface [4,5]. Histologically, septa divide the tumor into lobules composed of cords of spindled-to-oval cells embedded in a myxoid matrix. Some cases are hypercellular, with a rhabdoid or epithelioid phenotype, cytologic atypia, and minimal myxoid stroma, which can resemble other types of soft tissue tumors. The immunophenotype shows positivity for vimentin (75-80%), enolase (50-95%), EMA (10-15%), S-100 (15-20%), synaptophysin (40-50%), and INI1 [1,6,7]. The diagnosis is based on clinical, radiological, and histopathological features. Characterization of NR4A3 gene rearrangement is considered a desirable diagnostic criterion according to the WHO [8].

## Case presentation

**Figure 1:**
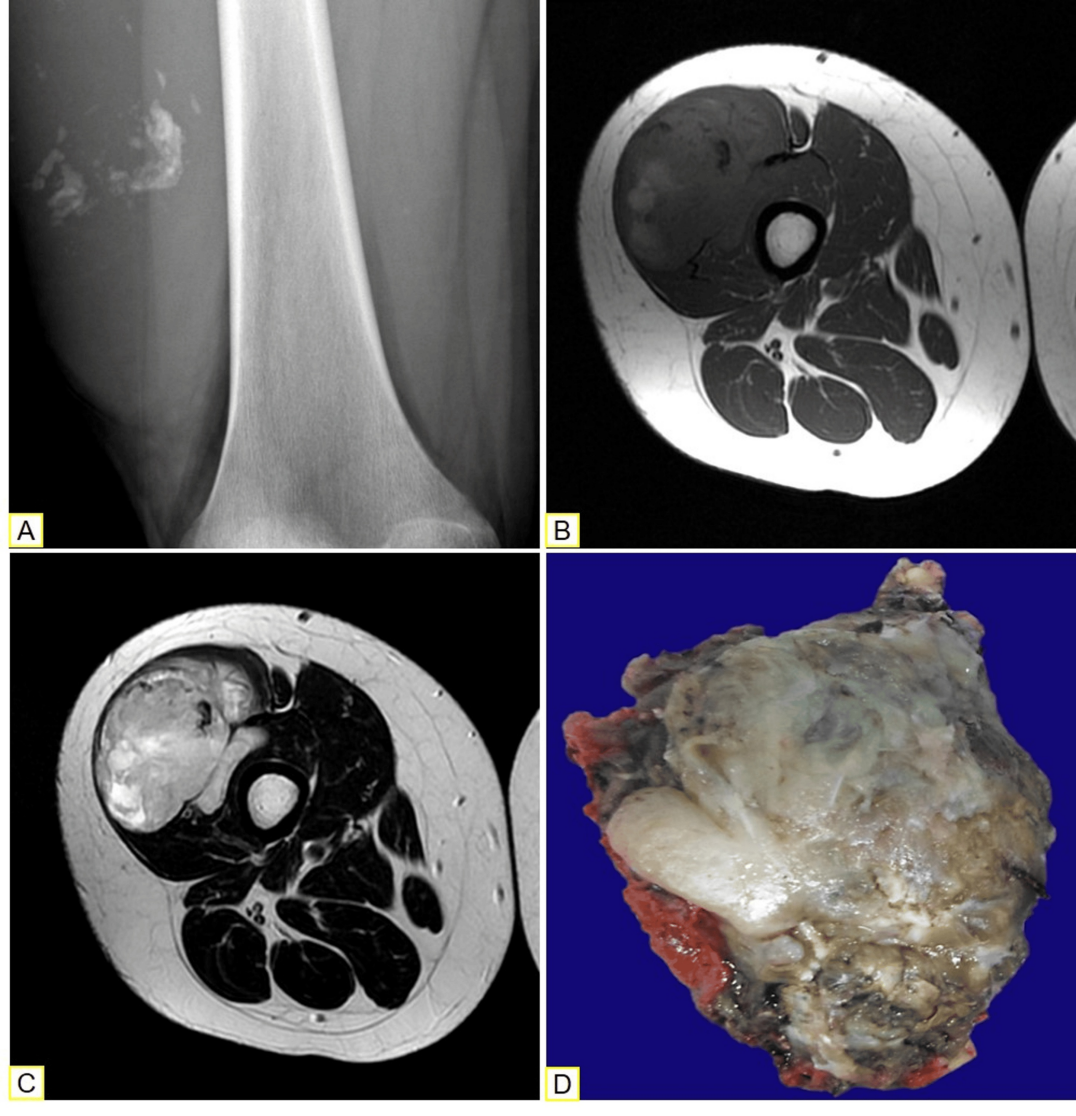
Morphological analysis. (A) Radiographic studies show a soft tissue lesion with amorphous, punctate, and curvilinear calcifications, suggestive of chondroid matrix. (B) The T1 sequence of magnetic resonance imaging shows a lobulated tumor with hyperintense (hemorrhage) and hypointense (calcification) areas. (C) Hyperintense areas (myxoid) in T2. (D) Macroscopic examination of the resection specimen reveals a lobulated tumor with cartilaginous and myxoid areas.

A 12-year-old girl presented with increased volume in her right thigh of three months’ duration; she had no other signs or symptoms. The skin overlying the lesion was erythematous. On examination, the lesion was hard and painful. It measured 13 × 17 cm. Radiography showed a soft tissue tumor with a chondrogenic pattern, without extension to bone. MRI revealed a heterogeneous tumor within the vastus lateralis muscle (Figure 1 A-C). 

**Figure 2:**
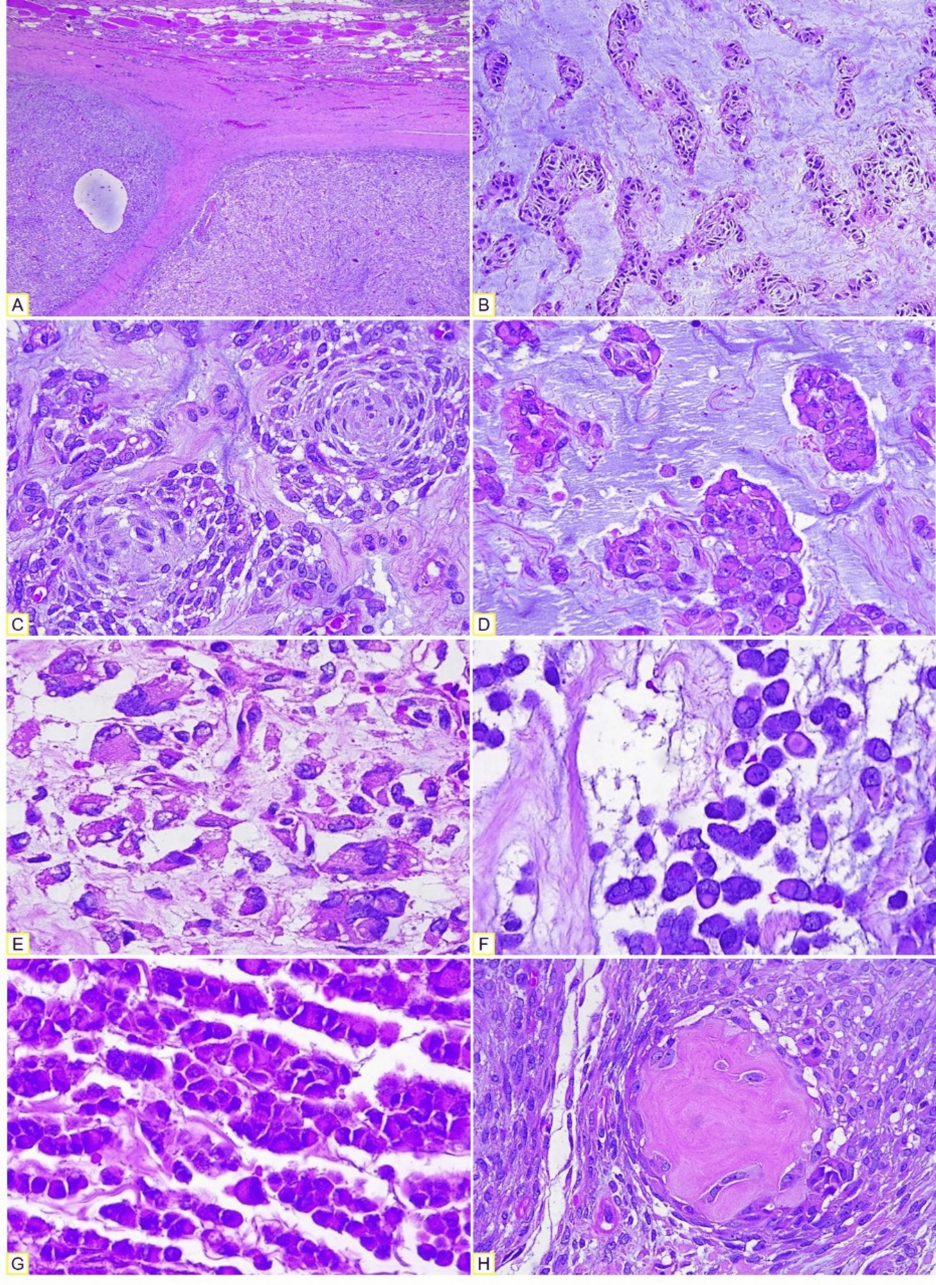
Microscopic examination (H&E). (A) Fibrous septa separating the tumor into lobes. (B) Anastomosing cords of neoplastic cells. (C) Primitive chondroblasts forming cannonball bodies. (D) Myxoid matrix. (E) Pleomorphism and high-grade atypia. (F) Rhabdoid cells with paranuclear hyaline inclusions. (G) Epithelioid cells forming cords. (H) Abrupt chondroblastic ossification.

The biopsy showed a malignant mesenchymal neoplasia with cords and individual cells of histiocytic appearance. Immunohistochemistry (IHC) was performed, showing positivity for S100, CD99, FLI1, and BCL2. Based on these findings, synovial sarcoma, rhabdomyosarcoma, lymphomas, vascular tumors, and histiocytosis were excluded. Five weeks later, the tumor was excised. The nearest distance to the surgical margin measured 1 mm. The specimen consisted of an ovoid tumor measuring 10 × 11.5 × 7.5 cm and was partially covered by fibromuscular tissue. The cut surface was heterogeneous and lobulated, with a myxoid appearance and hemorrhagic and necrotic foci (Figure 1D). Histologically, it was composed of lobules separated by fibrous septa with primitive chondroid cells (Figure 2A).

The neoplastic cells formed anastomosing cords that ended in small aggregates of cells organized in concentric whorls embedded in abundant myxoid stroma (Figure 2B-D). Some fields showed undifferentiated cells with vacuolated cytoplasm, pleomorphism, and multinucleated giant cells (Figure 2E). Others showed rhabdoid cells with paranuclear hyaline inclusions, as well as strings of epithelioid cells and osseous matrix with concentric configuration (Figure 2F-H). Likewise, some fields showed nodules of small round blue cells with abundant mitoses, fine branching capillary networks (also observed in liposarcomas), and zones of ossification.

**Figure 3:**
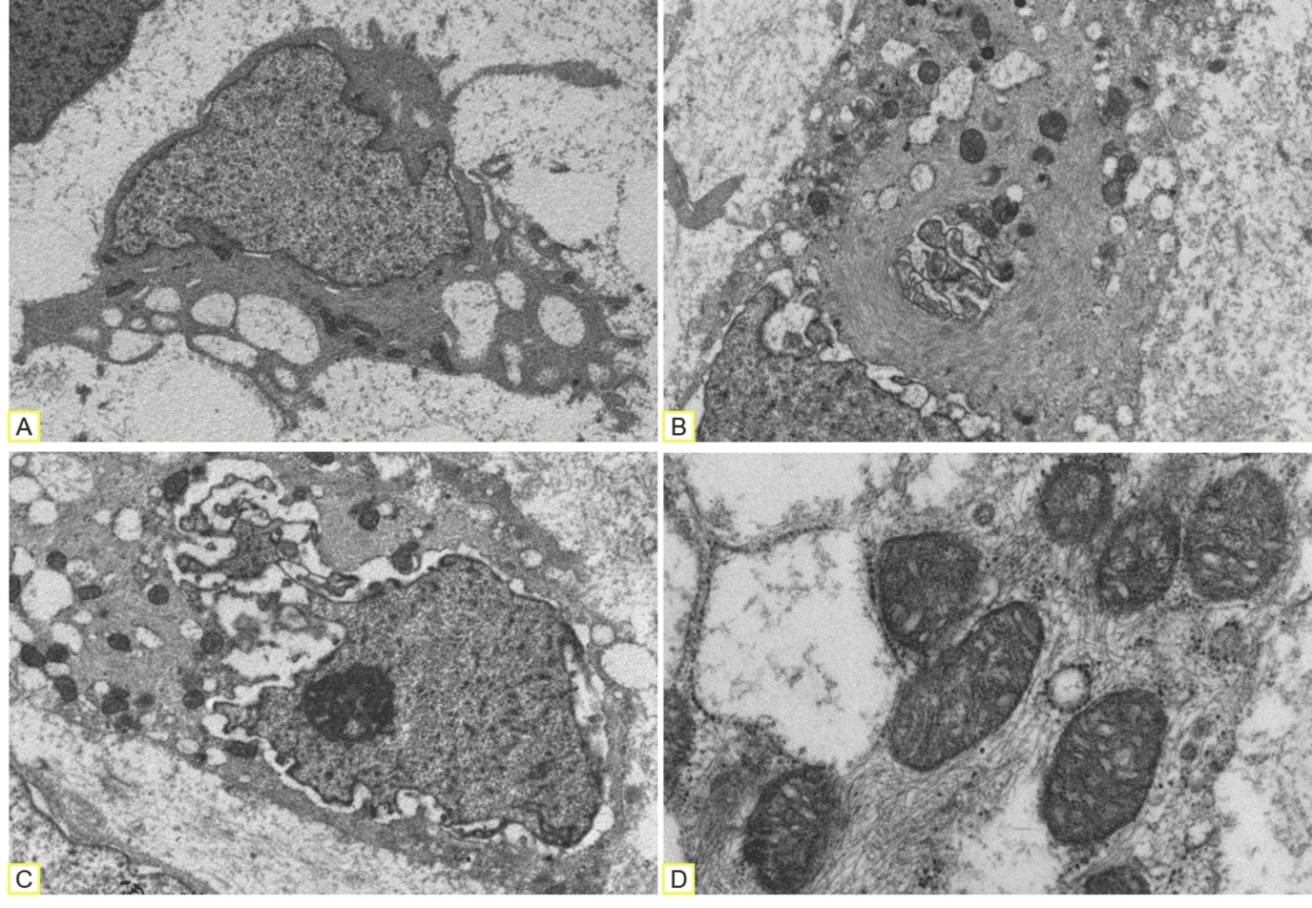
Ultrastructural examination. (A) Digit-like cytoplasmic projections. (B) Paranuclear swirls of intermediate filaments. (C) Distinctive nucleolus and well-developed Golgi complex. (D) Numerous mitochondria and prominent rough endoplasmic reticulum.

IHC showed positivity for S100, vimentin, INSM1, and INI1. Electron microscopy was performed, showing cells with digitiform cytoplasmic projections, paranuclear swirls of intermediate filaments, well-developed Golgi complexes, prominent endoplasmic reticulum, and numerous mitochondria (Figure 3A-D). With these findings, the presence of primitive chondroblasts was corroborated. The diagnosis of an EMC with rhabdoid differentiation was made. 

**Figure 4:**
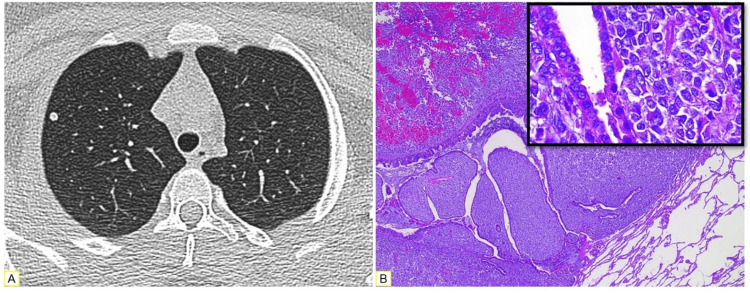
Characteristics of the metastasis. (A) A thoracic CT scan revealed a nodular lesion in the upper lobe of the right lung. (B) Microscopic examination confirmed extraskeletal myxoid chondrosarcoma metastasis.

Upon staging, a right pulmonary nodule was identified (Figure 4A). After excision, we received a 9 mm lesion; the histological sections demonstrated metastasis of EMC (Figure 4B). 

The patient presented with a deep tissue infection of the surgical wound. Once resolved, she completed chemotherapy with isophosphamide and doxorubicin, as well as radiotherapy with a total of 60 Gy. She relapsed locally sixteen months after treatment. The tumor was again excised with negative margins. She developed a deep tissue infection with osteomyelitis. She was to receive radiotherapy after completing treatment for the surgical site infection but was lost to follow-up.

## Discussion

In 1953, Stout proposed the term “extraskeletal chondrosarcoma” when reporting seven cases of the neoplasia in soft tissue without evidence of bone lesions [9]. In 1972, Enzinger and Shiraki described thirty-four cases with abundant myxoid matrix; they identified that this characteristic was associated with a better prognosis. Therefore, it was defined as a different clinicopathological entity [1]. Two years later, due to the resemblance of the primitive chondroid cells to embryonal cartilage, Albores et al. proposed the name “embryonal chondrosarcoma”; nevertheless, the term EMC prevailed over the latter [10].

Rhabdoid characteristics in EMC have been identified in 3% of reported cases in the literature [6,11,12]. Several studies have found an association between this morphology and increased aggressiveness, similar to the behavior reported in other neoplasias with rhabdoid differentiation [13-17]. Furthermore, it has been established that most EMCs with non-EWSR1-NR4A3 fusion genes (TAF15, TCF12, TFG, FUS, and HSPA8) have a rhabdoid phenotype, higher-grade morphology, and worse prognosis when compared to those with the EWSR1-NR4A3 fusion gene.

Despite the essential diagnostic criteria for EMC being morphological, in some cases, they are not sufficient to differentiate EMC from other neoplasias. Therefore, characterizing their immunophenotype is of great help [6,18]. According to the WHO, there is no definitive evidence of cartilaginous differentiation. Nevertheless, several studies have shown cartilaginous features in the tumor through ultrastructural analysis and immunohistochemistry [14,19].

The NR4A3 rearrangement was not evaluated in this case. However, it is not a prerequisite for establishing the diagnosis [18]. In fact, in some cases, the fusion gene EWSR1-NR4A3 cannot be identified, suggesting genetic heterogeneity in the lesion [2,3,18]. Moreover, there are non-EWSR1-NR4A3 fusion genes that can only be evaluated via next-generation sequencing [8,20].

## Conclusions

We report a case of extraskeletal myxoid chondrosarcoma with rhabdoid differentiation. Due to the lack of accessibility to characterize the NR4A3 rearrangement, the integration of clinical, radiological, histopathological, and ultrastructural data was sufficient to establish the diagnosis. This case exemplifies the aggressive behavior associated with EMC with rhabdoid features, confirming its poor prognosis.
